# What Mary Toft Felt: Women’s Voices, Pain, Power and the Body

**DOI:** 10.1093/hwj/dbv029

**Published:** 2015-09-23

**Authors:** Karen Harvey

**Affiliations:** University of Sheffield

## Abstract

In autumn 1726, Mary Toft began to deliver rabbits in Godalming, Surrey. The case became a sensation and was reported widely in newspapers, popular pamphlets, poems and caricatures. Toft was attended by at least six different doctors, some members of the Royal College of Physicians or attached to the Royal Court, but no doctor declared the affair a hoax until Toft herself confessed on 7 December 1726. This article focuses on Toft’s three surviving confessions in order to explore not the doctors or even wider representations of the affair but instead the person of Mary Toft herself. These rare sources give rare insight into one woman’s experiences of reproduction in the early eighteenth century. The essay engages with recent work on recovering women’s voices in the past, reconstructing Mary Toft’s words and her embodied and affective experience of the affair. These documents suggest a revision to our understanding of the hoax of 1726, one that situates the affair not in the context of the scientific revolution and Enlightenment or the assumption of men’s control over midwifery, but instead in the context of power dynamics amongst women in the practices of early-modern reproduction and birth.

## ***

On Monday 10 October 1726, the *Weekly Journal or British Gazeteer* published the first newspaper notice about the case of Mary Toft:

From Guildford comes a strange, but well attested piece of News. That a poor Woman who lives at Godalmin, near that Town, who has an husband and two Children now living with her; was, about a Month past, delivered by Mr. John Howard, an eminent Surgeon and Man-Midwife living at Guildford, of a Creature resembling a Rabbit.

The notice provided what became the standard explanation for the deliveries until it was finally exposed as a hoax two months later. That explanation came from the mouth of Mary Toft herself:

The Woman hath made an Oath, That 2 Months ago, being working in a Field with other women, they put up a Rabbit; who running from them, they pursued it, but to no purpose: This created in her such a longing to it, that she (being with Child) was taken ill, and miscarried; and from that Time, she hath not been able to avoid thinking of Rabbits.^1^

This version was repeated by Mary Toft again in November, when she was questioned by one of the many doctors involved in the case. Nathaniel St. André gave his report of this exchange in the first of many pamphlets published on the case. Toft’s ‘account’ was given in response to his ‘several Questions’. She explained that on 23 April, she was weeding in a field with other women when she chased a rabbit and ‘this set her a longing for Rabbets, being then, as she thought, five Weeks gone with Child.’^2^ The women ‘charg/ed her with longing for the Rabbet they cou’d not catch, but she den’y it’.^3^ She then dreamt of rabbits and had a ‘constant and strong desire to eat Rabbets, but being very poor and indigent cou’d not procure any’. Seventeen weeks later (towards the middle of August), ‘she was taken with a Flooding and violent Cholick Pains, which made her to miscarry of a Substance that she said was like a large lump of Flesh’.^4^ Three weeks later (early September) she passed another substance, though she continued to exhibit ‘the Symptoms of a breeding Woman’. At this time, as she worked in a hop ground, milk ‘flow’d profusely from her Breasts’ though, she added, ‘as she had Children before, she thought she felt very differently from what she used to do’. Finally, on 27 September, she was taken very ill, sent for her mother-in-law (‘who is a Midwife, and a neighbouring Woman’) and finally ‘voided’ what she described as parts of a pig.^5^ Following the delivery of further animal parts, she was churched ‘and thought all was over with her’.^6^

**Fig. 1.**
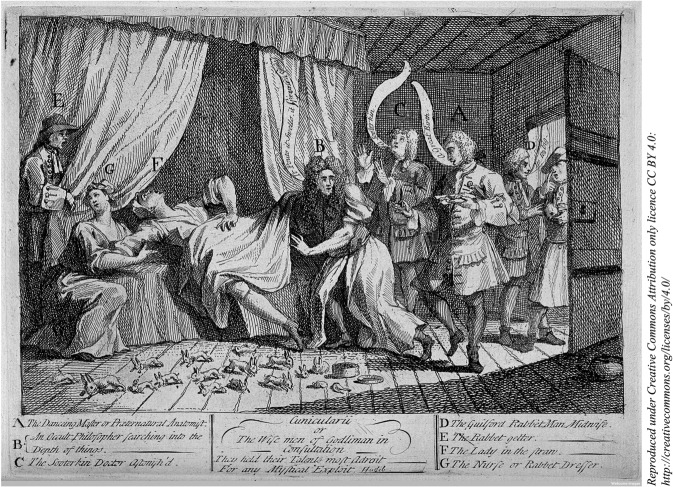
William Hogarth, *Cunicularii, or the Wise Men of Godliman in Consultation*, Etching, 22 December 1726. Wellcome Library, London [17342i].

Mary Toft continued to deliver rabbits throughout the autumn of 1726. In Godalming, and later in London, Mary Toft was attended by at least six different doctors, some members of the Royal College of Physicians or attached to the Royal Court, but no doctor declared the affair a hoax until Toft herself confessed on 7 December 1726. The case became a sensation and was reported widely in newspapers, popular pamphlets, poems and caricatures. There was a rich vein of prurience running throughout, but depictions of Mary Toft herself shifted quickly in tone from mildly sympathetic curiosity to indignant outrage. Most famous in this flood of print was William Hogarth’s 1726 engraving, *Cunicularii, or the Wise Men of Godliman in Consultation* (Fig.1). Hogarth stages the hoax as a drama performed by Toft to a group of elite men (including four of the doctors) with a supporting cast of her husband and sister-in-law. The engraving mocks the doctors for their credulity.^7^ So whilst we might detect in Hogarth’s parody of the Adoration and Mary’s delivery of Christ some sympathy towards Toft, the image is premised on the principle of Mary Toft’s volition. Ultimately, Toft was portrayed as a devious woman who set out to hoodwink several doctors and make her fortune. That she might do so reflected the longevity of classical beliefs in the possibility of monstrous births caused by the maternal imagination. Women’s thoughts – in particular their thwarted desires – could affect their unborn in physical ways.^8^ As we have seen, this was the explanation given by Toft herself and it guaranteed the keen interest of the doctors. This context, combined with the rich printed sources generated by the doctors involved, has meant that the case has been studied most often from the perspective of those doctors or in the context of medical or wider knowledge about reproduction, particularly the growing authority of (masculine) medical authority over reproduction.^9^ The case has been presented as a hangover of an earlier, more superstitious, society, with its unmasking as a hoax itself helping to propel knowledge of reproduction and childbirth into a more rational age.^10^ More recent works show how repeated invocation of the case throughout the eighteenth century suggests the continuing power of the prodigious.^11^

In this essay I will take a different approach. My focus here is not on the doctors or even wider representations of the affair but instead on the person of Mary Toft herself. The description of Toft in the *Oxford Dictionary of National Biography* is terribly short: ‘Mary, an illiterate, was of small stature, with a healthy, strong constitution, and a sullen temper.’^12^ Though not acknowledged in the entry, this is a paraphrase of the description of Toft in St. André’s pamphlet: ‘She seem’d to be of a healthy strong Constitution, of a small size, and fair Complexion; of a very stupid and sullen Temper: She can neither write nor read’.^13^ Rather than rely on such printed accounts, I will try to reconstruct Mary Toft’s experiences using the richest (and arguably most difficult) documents we have for the case: the thirty-six pages of rough copies of three ‘confessions’ or statements given by Mary Toft in December 1726.^14^ For so extraordinary an episode, it is remarkable that no historian has attended closely to these statements given by the protagonist. As Lisa Cody has demonstrated, in the absence of clear medical proof, the several doctors involved in the case instead relied upon the word and regarded Mary Toft as ‘the sole authority and author of fact’.^15^ Cody acknowledges that Toft got nothing from the hoax and hints that she was ruled by others, but regards portions of Toft’s confessions as ‘probably fabricated’ and concludes that we ‘will never really know how or why Mary conceived her project’.^16^ Dennis Todd dismisses the confessions as a simple exercise in dissembling: the hoax was ‘motivated solely by money’, he claims, and Toft must have been ‘devoted’ to it.^17^ This essay offers a different interpretation. Undertaking a close examination of the confessions, I will engage with recent work on recovering women’s voices in the past. While the confessions do not offer direct access to Toft’s motivations, and even less a neat description of the events that took place, I argue that we can reconstruct Mary Toft’s words. I then show how we can use the confessions to reconstruct her embodied and affective experience. There is a range of possible explanations for the hoax, though it is likely that Mary Toft’s expressions about pain and emotion in the confessions provide an answer to the question that the approaches of intellectual, medical and cultural historians have so far obscured, and that is ‘what really happened?’ Nonetheless, the confessions certainly make accessible to us Mary Toft’s personal truth about her physical and subjective experiences. They are incredibly valuable in themselves as rare sources that give insight into one woman’s experiences of reproduction in the early eighteenth century. More particularly, the confessions reveal a series of events much darker and more disturbing than the fluffy bunnies hopping in the foreground of Hogarth’s print. These documents suggest a revision to our understanding of the hoax of 1726, one that situates the affair not in the context of the scientific revolution and Enlightenment or the assumption of men’s control over midwifery, but instead in the context of power dynamics amongst women in the practices of early-modern reproduction and birth.

## MARY TOFT’S VOICE

Mary Toft was born on 21 February 1703 to John and Jane Denyer. In 1720, aged seventeen, she married eighteen-year-old Joshua Toft, a wool cloth-worker. They were both young for marriage in a society where the average age of marriage was nearer twenty-four, although couples of their non-skilled rank often married at a younger age.^18^ Their first child Ann was born on 27 March 1723, though she appears to have died in July of that year. The birth of their son James followed twelve months later on 8 July 1724. Thus, at the time of the hoax Mary had given birth to two children with one still living. A third and final child, Elizabeth, was christened on 4 February 1728, a year or so after the rabbit affair. Mary’s parents, John and Jane Denyer, had five children of whom Mary was the second eldest. She was, though, the oldest girl; this may be why she left home to marry so early, though it is also possible there may have been an earlier pregnancy. Her parents are entirely missing from the story of the case, typical of the severing of these connections when a woman married, though the absence of her mother is perhaps peculiar given the nature of events. Joshua Toft, Mary’s husband and senior by only two or three months, was the sixth of twelve children. Joshua was named after his father and also shared this name with his elder brother, his parents’ first son, who had died two years before Joshua (jun) was born.^19^ Such naming practices were not unusual and reflected the importance of lineage and the family community. Mary Toft’s miscarriage of spring 1726 and the animal births that followed thwarted the powerful impulse to reproduce this family.

The early newspaper accounts of the case and the account given by Mary in St. André’s pamphlet were produced when Mary was in Godalming.^20^ She was subsequently moved to the town of Guildford and then to London, where she came under the closer observation of the doctors and also of men representing the criminal justice system. From the 29 November 1726, Mary was lodged at Roger Lacy’s bagnio. A porter of the bagnio reported that rabbits had been smuggled in to Toft and this triggered the indictment of both Mary Toft and John Howard for the misdemeanour of imposture.^21^ This is why the confessions were produced. The first two statements were given on 7 and 8 December. The third was given on 12 December in the Westminster Bridewell prison, following Toft’s indictment for imposture. All three were taken down by the doctor James Douglas (in whose papers they now survive) and were part of a formal legal process given in the presence of the London Justice of the Peace, Thomas Clarges, as part of his criminal investigation into the hoax. This is why they were never published in their entirety, though Richard Manningham (another doctor present) published a short summary of the first confession in his *An Exact Diary*.^22^ As Douglas explained, he would not publish the confessions as the depositions were in the hands of a magistrate ‘to whom she has delivered her Confession ^ ^under oath^’ and were to be submitted to the court.^23^ Toft’s earlier accounts were also prefaced with an oath, as the extract from the *Weekly Journal* notes, but the three confessions at the heart of this essay marked the shift of the case from medical to criminal context.

Since Carlo Ginzburg explored the inquisitorial records for the cases of the Friuli miller, Menocchio, analysing in them both ‘the gesticulated, mumbled, shouted speech of oral culture’ and the ideas of elites,^24^ historians have become adept at using statements, depositions and confessions to access different – including ‘subaltern’ – voices, often through micro-histories.^25^ Historians are also practised at lifting women’s voices out of historical sources, early modern court depositions in particular.^26^ Frances Dolan has recently problematized these methods, though, by emphasizing the collaborative nature of such documents. Dolan is sceptical that historians can locate the agency or voice of the speaker because, ‘the I who speaks is often someone else’, shaped by the questions and expectations of others.^27^ The nature of Mary Toft’s three confessions is certainly less than straightforward. They were extracted under threat of ‘a very painful Experiment’ (uttered by Richard Manningham)^28^ and in the presence of five men (two of them titled), following weeks of frequent physical examination and round-the-clock observation with sometimes ten people (possibly more) in the room at one time. They were created partly in response to specific questions posed by Clarges, the Justice of the Peace. An added difficulty is that Mary Toft is trying to explain something that most now believe to be impossible, as in cases of witchcraft and possession.^29^ Moreover, the documents themselves are extremely messy. The awkward syntax, narrative structure, frequent markings, crossings out and irregularity in tense and pronouns are all written in the rushed hand of James Douglas as he tried to keep up with the fast-moving speech during interrogations that Manningham suggested were ferocious.^30^ There is no doubt these are heavily mediated documents that combine the different voices of the interrogator (Clarges), the amanuensis (Douglas) and Toft herself.

Yet these voices can be clearly distinguished. Beginning with Toft’s startling opening statement in the first confession – ‘I will not goe on any longer thus I shall sooner hang my selfe’ – several passages read more like oral transcripts than the seamless collaborative narratives that provoke Dolan’s scepticism.^31^ Whilst we know that tidied versions were sent to a court, most likely the Westminster Sessions, these three confessions are rough and unfinished documents. Such documents were not submitted to courts so these confessions are rare survivals that allow us insight into procedures of interrogation. They also give us access to Mary Toft’s words. Large sections of these documents are free-flowing descriptions from Toft, but they also contain clear insertions from the interrogator. There are notes that appear to be commentary from Clarges, such as ‘N:B: it seems they come to put up one piece after they had taken away another’.^32^ There are also indications of when Mary Toft was interrupted and asked a question. Some way into the first confession, for example, a horizontal line immediately following a comma after ‘eate’ on the line above indicates that both Toft and the note-taker have been interrupted. The next line begins, ‘She protests and declares’, and asserts Howard’s ignorance of the hoax, indicating that Toft has been challenged and asked a question about John Howard’s guilt, though she had not been talking about him at that point.^33^ Clarges seems to suspect Howard, then. The same line appears in the second confession. Toft begins the sentence ‘when all the monstr’ but breaks off and Douglas leaves a large ‘X’ as a place-marker and another horizontal line. Her response – ‘I am now asked to tell you the truth after I have told so many lyes’ – suggests she has been asked to tell the truth.^34^ It is significant that she is interrupted when she utters the word ‘monster’: by this stage (8 December) her interrogator knows the monstrous birth to be a lie.

The way that these confessions capture the dynamics of the interrogation and register the different voices is underlined by the two tidied versions of the first confession preserved in Douglas’s papers. These remove all evidence of the breaks in the interrogation, the questions and her direct responses. There are also some changes to the content. An unnamed woman who urges Toft to undertake the hoax says she must go through with it ‘now I had begun, but that she have / some of the money’.^35^ The second clause about the money is not in the original, however.^36^ The second tidied version of the first confession includes judgements that clearly belong to Douglas or Clarges. The nameless woman is now ‘the strange Woman’ acting ‘upon a frivolous pretext’.^37^ These revisions do not alter significantly the basic events as told by Toft in the rough confessions but these and other changes suggest that those original confessions were relatively unedited records of the interrogations. This is not to deny either that some of Toft’s recorded speech in these confessions may have departed from her actual spoken words or that her utterances were shaped by the questions posed by others. Nevertheless, we are able to distinguish with some confidence her words from theirs in the three rough confessions.

Even if we can confidently distinguish Mary Toft’s utterances from those of the interrogator, a problem remains over their status as truth claims. We know – as did her interrogator – that she *was* lying about the monstrous births, though this occupies a relatively small part of the first confession. Questioned as part of a criminal investigation, Mary Toft had ample reason to lie throughout. And as Cody points out, the three confessions are conflicting.^38^ Confession one blames the unnamed woman (a knife-grinder’s wife) for the hoax, while confessions two and three apportion blame to Toft’s mother-in-law and John Howard, the Guildford doctor who first attended the births. Yet there are remarkable consistencies in the content that are entirely superfluous to the investigation. It is striking that when we apply the technique of criteria-based content analysis (CBCA) used by forensic psychologists in their analysis of witness statements, Mary Toft’s confessions meet the criteria for a statement of memory not fantasy. As we shall see, they have a logical (not necessarily plausible) structure, are rich in specific detail, embed the events in an everyday context and describe affective interactions with others, for example.^39^ Toft’s confessions are a resounding fail on the final ‘validity checklist’ because her statements were made in coercive circumstances and therefore she had questionable motives in reporting.^40^ Yet the initial results using CBCA are suggestive. Being dishonest and strategic still requires the speaker to draw on her beliefs.^41^ There is truth in false report, in other words. The confessions contain details about Toft’s neighbourhood that are historically convincing and key features of the content remained constant even as she retold events between October and December 1726. These stable elements of the confessions, some of which I discuss in the following section, are Toft’s distinctive contributions to the documents.

Yet these confessions do not only give us access to Mary Toft’s words. Carlo Ginzburg’s view that inquisitorial records show oral culture as ‘almost an extension of the body’ is particularly pertinent, here, because the most striking feature of the three confessions is the urgency of Toft’s repeated descriptions of her body.^42^ These sources thus allow me to move past a cultural history of the body focused on discourse and instead to explore experiences of the body as communicated through language.^43^ Toft’s quickly spoken words are substitution for her body. The way she links body and mind in her expressions is also where we can locate the words that refer to her emotional state.^44^ Examining closely the structure and narrative of the confessions, the major themes of the confessions and the language that Toft reportedly used, we can observe how these documents convey Toft’s embodied and affective experience.

## PAIN, TOUCH AND POWER

Mary Toft describes two sets of events in her confessions. First, having already had two children (and having lost one of them), she suffered a miscarriage. The confessions provide rare evidence of a woman’s way of describing and making sense of this. The description in the first confession begins with Toft telling the story of the monstrous birth: ‘I was delivered of a furie monstrous / Birth. The liver and gutz came away first.’ She is then interrupted and presumably asked to explain how this came to happen. She then goes back further in time to recount the story of the miscarriage: ‘Some thing came away with a flooding / After I had seen some rabbits whc / I longed for.’^45^ She continues her description of the miscarriage and only returns to the liver and guts of the rabbit on the third page of the confession. For Toft, then, the miscarriage and the rabbit births were separate physical events and throughout ‘monstrous’ is a word she reserves solely for the later rabbit births. Her description of the miscarriage is lengthy and detailed. She describes how she saw the rabbits running away from her in a field, later passed an object ‘as big as my arm’ and then experienced her first ‘flooding’ that lasted a week. Then, three weeks later, she was working in a hop-garden a quarter of a mile from her home, when she experienced further flooding and great pain.^46^ Miscarriages can be prolonged events lasting several weeks and this is what Mary is describing. The second confession skips this detail, though it is repeated in condensed form in the third. All the confessions, though, begin with Mary’s description of flooding, her body being ‘open’ and passing fleshy objects. The descriptions are immediate and powerful and they draw on experiences she has had with other pregnancies: ‘The same day the waters came as in your other labours’, she says on 7 December. On the following day, she was ‘very ill of a flooding’ and ‘My body was so open as if a child had just come away’.^47^ Throughout the confessions, the chronology Mary Toft gives of her miscarriage (which had happened months before the interrogations) and the early stages of the rabbit births match very closely: she is precise and consistent about weeks, days, hours and times of day. The repetition of detail on the miscarriage is strictly unnecessary in explaining how the rabbit births began. Yet it is striking that despite being under threat to explain how the hoax took place, the story of the miscarriage is the one she wants to tell. As we have seen, it is a story she has been telling for weeks.^48^

Mary Toft’s insistence on this sequence of events throughout the autumn of 1726 shows determination and a sound memory. It establishes Toft’s authority as a narrator and suggests that claims that she was mentally deficient are unconvincing. The story is certainly a kind of fiction, though not one geared to the needs of her interrogator. Instead, the inclusion of superfluous details – details that her interrogators do not want to hear given that they are now aware of the hoax – might suggest a degree of compulsion on the part of Mary Toft. The narrative gives form and meaning to the events she has experienced. Toft was operating within a long-standing confessional culture but a growing interest in individual subjectivities within print culture was reflected in part in new forms of first-person narratives during the 1720s in which women gave (or appeared to give) autobiographical accounts.^49^ This affected the narrative structure of evidence, too.^50^ Toft’s insistence, in confessions one and three, on the miscarriage as a prelude to what follows situates her entire narrative firmly in the context of a real physical trauma. Alternatively we can say that Mary Toft’s confessions narrativize the events as trauma, ‘a language in which to speak of the wounds of the past’.^51^ Writing and speaking about physical trauma deploys narrative to exert control over a situation that otherwise threatens physical and mental disintegration.^52^ Toft’s descriptions of the miscarriage suggest her sense of a lack of control over her body. The verb ‘flooding’ used by Toft to describe both the miscarriage and the animal deliveries is one example; the fact that this word was used by many women to describe birth in this period suggests that Toft was relating her experiences of 1726 to her previous experiences of birth.^53^ Unlike witch confessors, Toft’s narrative gives her no volition and, as we will see, the verbs she uses ascribe agency to others.^54^

The second event and the main subject of the confessions is the hoax itself. Concerning these rabbit births, Toft is consistent on a number of details: her mother-in-law enters early in the story and this marks the appearance of the unidentified parts; one Mary Gill arrives to examine the parts Toft first passes into a pot; parts, including bones, are inserted into her body by someone else; and finally Toft is advised by someone else to insert the parts herself when she is alone in London. Though a hoax, it is important to note that Mary Toft did experience a sequence of real deliveries: animal parts were placed inside her vagina (even, according to one doctor, inside her uterus) and they exited her body.^55^ This was a difficult process. St. André reported the early pieces of animal bodies in some detail (he is more interested in these animal bodies than in Toft’s). The first part he delivered was, ‘the entire Trunk, strip’d of its Skin, of a Rabbet of about four Months growth’.^56^ A later description reads, ‘The Nails of the Paws were most of them exceedingly sharp’.^57^ Such parts, and many others like them, were hidden in Mary’s body over a period of several weeks. She must have become very ill and was surely lucky not to have died. The hoax was constituted from this real physical process.

It is therefore hardly surprising that the most repeated comments in Toft’s confessions refer to pain (seventy-one references in total across the thirty-six pages). Mary’s descriptions of pain are useful for the historian because they allow us to access her embodied experience of the situation. Pain is both a psychological and physiological phenomenon and expressions of pain reflect (and can therefore tell us about) the context or conditions in which the experience of pain itself took place.^58^ Expressions of pain also connect to the physical sensation of pain because ‘major kinds of pain are characterized by distinct constellations of words’.^59^ It is for this reason that patients’ descriptions of pain are reasonably reliable diagnostic indicators.^60^ For historians, too, pain is a way to study the physical and cultural body.^61^ Most of Mary’s references to physical pain in the first confessions simply state the fact of pain, as in her comment ‘gt pains all that day’.^62^ Mary layers pain over pain for emphasis: ‘after Mr. Howard came / away some thing still coming & pain in pain / all that time and not able to goe across / the chamber. Two dayes after the 14.N. was / out one foot came away with a great pain’.^63^ Phrases such as ‘the night I was taken with violent pains’, ‘then in great pain and floodg’, and ‘being in gt pain’ appear to serve no specific narrative purpose and give no specific cause.^64^ This is simply pain. It is likely that Toft exaggerated her pains in an attempt to attract sympathy. Indeed, on two occasions she refers to ‘feigned’ pains: ‘feigning great pains’ to attract John Howard in the first confession and in the third confession explaining how her ‘feigned pains’ were to bring down an animal part.^65^ These two references are flanked by many other descriptions of apparently real pains, however. For example, the third confession also contains the statement, ‘a gt many pains had brought it down’.^66^ Given that by this stage Toft was incarcerated in the Bridewell, Toft could have been honest about all her pretended pains. Her repeated unqualified references to pain show that Mary Toft did indeed experience extreme pain during the two months of the hoax.^67^ This necessarily adjusts our view of her as simply a dissembler: this was a genuine physical trauma and would have been extremely difficult to execute alone.

Many of Mary Toft’s references to pain describe it as functional. In the first confession she reports that her mother ‘said that every pain would bring it forward’, and this is what then appears to happen.^68^ These descriptions are grounded in contemporary discourses of pain rooted in the humoral theory in which bodies experienced flow and movement within, as well as according with classic labour pains caused by the contraction of the uterus.^69^ Rarely, Toft uses metaphors to express her pain. She reports, **‘**I told M^r^ Howd that I had pains that last night like the tearing of brown paper’, a metaphor she had used in her earlier interview with St. André.^70^ In the second confession she describes ‘a pain like a pricking of bones within me which continued for an hour or more’ and pains that felt like ‘a great forcing down that I could scarcely bear it’.^71^ Toft describes being torn or ripped apart. Perhaps not surprisingly, this is a common trope in both early modern and modern descriptions of the pain of childbirth but Toft’s personalization of these common metaphors and her deviation from typical idioms are revealing. In women’s descriptions of pain in childbirth, the instruments responsible for the tearing or splitting have changed over time: for Alice Thornton writing in the seventeenth century it was the rack and she adopted the role of martyr; for modern mothers it is often metallic mechanical parts, particularly if the event is taking place in a hospital.^72^ On just one occasion, in her final confession given in the Bridewell, Toft uses one of these specific metaphors, declaring, ‘(I was all n.[ight] in a most violent Rack and torture)’.^73^ The comment comes at the end of a long section that describes in gory detail the jagged pieces of bones and skull that Toft’s mother-in-law has put inside her body. Her pain is not caused by a disembodied instrument of torture. In this context it is also significant that other common elements of early-modern birthing women’s descriptions of pain are missing from Toft’s words. The Providential narrative, with its concomitant godly suffering and torture of Christian martyrs, is notably absent.^74^ Toft allows no role for God in her experience of pain. If earlier portents were prophetic of God and the natural world, by 1726 the emergence of new explanatory systems meant that events such as monstrous births were rarely perceived as prodigies and if they were this was decried as ‘superstition’.^75^ This does not mean that the Toft case was not read for hidden import: it was viewed by some as a targeted political comment on the court of George I, for example.^76^ In Toft’s confessions, though, this hoax is explained not by the natural world or politics but by human action. Elaine Scarry finds that though itself inexpressible, when pain finds a voice it starts to tell a story.^77^ As each of Toft’s narratives moves forwards, her pains are caused by others. In the first confession the instigator is the wife of the knife-grinder; in the second and third confessions the pain is caused by Howard and her mother-in-law. They wait for her to experience pain and then both extract and insert animal parts at those moments. This is surely why she is afraid of being touched: ‘I was loath She should touch me’, she says of her mother-in-law in the final confession.^78^ Toft’s use of the verb ‘touch’ is revealing. Several people ‘put’, ‘take’ and ‘bring’ animals, but ‘touch’ refers specifically to an examination. In the second confession, for example, Toft reports how St. André touches her and then takes something away.^79^ The consistency of Toft’s descriptions of pain and its cause couched in specific but historically authentic language suggests that these events are real and are being done to her.

## EMOTIONS AND FEAR

Toft’s descriptions of physical pain are also indicative of her emotional or psychological states. Her description of herself as ‘very uneasie’ refers to pain, whereas ‘easie’ is counterposed to being ‘greatly in pain’.^80^ St. André described Toft as ‘chearful and easy’ after the delivery of animal parts, noting that she herself reported being ‘tolerably easy’ at these times.^81^ In the second confession she says she allowed herself to be moved to Guildford because, ‘I thought my selfe in a very desperate condition in hopes of relief’.^82^ Here, Toft refers to her physical condition (which needs relief) in more general terms (a very desperate condition). Emotions are embodied in social and physical practices and this body-emotion connection was highly gendered in the early modern period. It is difficult to disentangle a woman’s description of physical and emotional anguish because women in particular linked their experience of emotions (their own and other people’s) to direct effects on their bodies.^83^ Toft’s body – her physical suffering – expressed her emotional distress.

Reports on the case also attend to Toft’s pains and observers believed these to be genuine: St. André described ‘violent Labour-Pains’ so strong ‘that four or five Persons cou’d hardly confine her to an Arm-Chair’.^84^ For the doctors, pain was the only observable evidence of the hidden events taking place inside Toft’s body. So it is that examinations take place during her pains. Manningham announced to the ‘many Persons of Distinction’ in the bagnio that ‘if there was any Person present willing to examine her, that they would do it then while her Pains were upon her. Accordingly, several Persons did examine her’.^85^ Yet there is an almost complete lack of interest in Toft’s experience of pain. St. André comments on her ‘exquisite torture’, though he does nothing to alleviate it.^86^ The lack of interest in Toft’s emotional state is particularly puzzling given that this could have been fertile ground upon which to establish her dissembling. Yet only one person appears to have considered emotional response as a form of evidence: Mary Costen, the nurse to Mary Toft until she went to London and one of the witnesses whose statements were delivered to Baron Onslow as part of the criminal investigation of the case in Surrey. She suspected Toft’s husband Joshua Toft because he showed insufficient concern for his wife: ‘this Deponent never saw him dejected, or any ways concerned for his Wife’s Misfortune’.^87^ Already a defendant’s guilt or innocence could be judged partly on their emotional responses and mental incapacity could be used as grounds for a defence of diminished responsibility in cases of infanticide.^88^ Yet those investigating the Toft case, whether as a medical or a criminal act, were not interested in her suffering. Toft’s insistence on pain is striking given the apparent disregard amongst the doctors for her suffering; again, this is something she seems compelled to describe. The emphasis on her own anguish was perhaps motivated by a desire to exculpate herself, perhaps by underlining her own victimhood, but it was also the result of the ongoing physical and emotional trauma of the hoax.

The lack of concern for Toft’s condition is also observable among the many women gathered around Mary Toft throughout the affair. Toft describes how early in her miscarriage women offered her support, working for her to allow her to leave the hop-garden without losing her pay.^89^ Yet otherwise, the kin and neighbours who surround her show no sympathy or concern. Toft mentions several women in her confessions: Mary Gill is reported as witnessing the first animal births, Mrs Mebbin removes a foot and Betty Richardson is at one point forced to remove an animal part from her body because Toft was in such pain.^90^ Most prominent is Mary Toft’s mother-in-law, Ann Toft. Mary is clear that Ann is involved in the affair from the start. Ultimately, it is Ann that Mary accuses. Restating her innocence and ignorance midway though the second confession, she is interrupted, asked a question and her reported response is unequivocal: ‘She is of opinion that if the Rabbits did not breed in her that her mother in Law and M^r^ Howard must have put them up her for nobody else came near her’.^91^ When asked in the final confession, given in the Bridewell away from her family and neighbours, why she did not tell the truth about her mother-in-law she responds, ‘I was very unwilling to tell the truth because it light upon she’.^92^ It was Ann, Toft deposed, that ‘told me that I would do it and go thro’ I should get a good living and be ruled by her and not tell of her’.^93^ Ann, Toft explained, ‘persuaded’ and ‘ordered’ her.^94^

The presence of the group of women that Mary describes in her confessions are verified by references to the shadowy women that operate on the periphery of the male doctors’ vision. When St. Andre first meets Toft she is ‘sitting on the Bed-side with several Women near her’.^95^ In his pamphlet, Manningham reported that the movement of Mary’s body was so violent during her pains that, ‘as I sat on the Bed in Company with five or six Women, it would sometimes shake us all very strongly’.^96^ It is testament to the usual presence of women at the bedside of birthing women that their names are not recorded, perhaps. But Toft gives information on them. Betty Richardson was a silkstocking-maker’s wife, Mrs Mebbin was a gentlewoman and Mary Gill appears to have been sent for to inspect the animal parts; all were described as neighbours.^97^ Ann Toft is consistently described as a figure of authority. When the animal parts first appear she takes the pot from Mary Gill and ‘shewed it to all the women who were not able to make any thing of it’.^98^ Toft repeats this scenario in the third confession, describing how Gill ‘carried it to my Mother who showed it to every body abou’t and seemed greatly surprized’.^99^ Ann Toft is repeatedly named as a midwife, though she is not one of the three women listed as midwives in the parish registers nor does she appear in the Bishop’s licences for midwives for the area.^100^ Ann Toft certainly had extensive experience of pregnancy and labour herself, having had twelve children over twenty-three years and she may have served as midwife to poorer women in the town. In contrast, in the spring of 1726, Mary Toft had already lost one child at three months, had a son rising two years and was experiencing a long miscarriage of a third pregnancy. In this context Ann Toft must have appeared a formidable woman. The actions of these women are certainly authoritative. Like the male doctors, they are described as ‘touching’ (or examining) Toft: ‘Then he [Howard] desired M^rs^ Mebbin ^a gentle w^ to touch me and she took away a foot’.^101^ This underlines the women’s competency and status. Such descriptions bring to mind not just the female-only lying-in ritual of this period but specifically seventeenth-century bedside conflicts and ‘the politics of touch’ around the body of the birthing mother reconstructed by Laura Gowing. In this environment, older and experienced female kin and neighbours practised ‘the intuitive, experienced touching that was identified with midwives and sexually knowledgeable women’, establishing their authority over younger women.^102^ From Ann Toft’s perspective, her young daughter-in-law may not yet have fulfilled her reproductive purpose. Female rivalries were certainly played out in cases of witchcraft and violence.^103^ At Mary Toft’s bedside these women are not only complicit in but appear to direct the hoax. None of these women offer her assistance and their actions have an altogether more sinister tone.

Rather than marking a change in Mary Toft’s story, the incrimination of Ann Toft in the second confession invites a reinterpretation of the first confession. It was here that Mary gave the account of the knife-grinder’s wife. Following her miscarriage, and the passing of several strange parts, she says, ‘a woman whom I don’t know if I was to be put to death’ came to find her brother to grind a stone.^104^ Unlike the other women Toft describes, this nameless woman was not a neighbour: she and her husband travelled ‘about the country’. After seeing Toft in pain and being told about the early animal deliveries, this woman suggested the hoax to Toft: ‘I told her such a thing would not be done. She sayd it could and desired to try’.^105^ The woman wanted money and Toft gave her three half crowns, apparently for the rabbits the woman procured.^106^ What follows is perhaps the most difficult account of the affair, with Toft the victim of this woman’s abuse. As the nameless wife of a knife-grinder, she is connected to a suitably intimidating occupation and herself wields a knife with which she cuts the dead animals. Lisa Cody describes this as an account of ‘essentially a bizarre sexual assault with a foreign object’, and one probably designed to protect herself and her family.^107^ Yet the theme of being abused by a sinister woman to whom she or her family is somehow obligated is consistent in Mary Toft’s narratives about the ordeal. What changes is that this shadowy nameless woman is replaced by the figure of her mother-in-law. The knife-grinder’s wife is a negative image of Ann Toft, unknown and distant from the family rather than at its heart. This takes us some way from the apparent certainties of social history and into an altogether more difficult area of unspoken emotions and family dysfunctions. Historians, particularly historians of witchcraft, are now adept at reading such narratives to tease out the social and emotional truths and community dynamics located in the stories of confessors. Fairies, for example, were a device that allowed women to express the complex emotions around child health and the loss of a baby.^108^ Women’s actions in witch-hunts were a way for them to describe or express traumatic events and fear of abuse and could reflect a profound ambivalence towards mother figures.^109^ The affair of 1726 has not generated such rich archives. Yet it seems incontrovertible that the knife-grinder’s wife was a figure that allowed Mary Toft to articulate women’s violence towards her at a time when, as Garthine Walker notes, ‘[n]o accepted concept of sexual threat existed to underscore women’s vulnerability at the hands of other women’.^110^ This figure was not perhaps a direct substitute for the person of Ann Toft, but rather embodied menacing women generally and the fear they provoked. In her story, Mary Toft expressed that she was not the perpetrator but the victim, and a victim at the hands of other women.

## CONCLUSION

Fear is a thread that runs throughout Mary Toft’s confessions. Though we know that women were sometimes frightened of childbirth and of monstrous or abnormal births in particular, Toft does not express fear of the miscarriage.^111^ Grief for the loss of this third child perhaps affected her state of mind and shaped the whole affair, though this is not clear from her confessions. She is certainly frightened of being touched. Yet when Toft uses the word ‘affrayed’ she refers to the fear of being exposed.^112^ Richard Manningham’s most striking comment on Toft’s distress is revealing: he reports that she cries just once and that is when he tells her he does not believe her story.^113^ We can well imagine why this labouring woman was frightened of being found out in the presence of the several high-ranking men in her room. But the repeated details in her narratives indicate that she was frightened of something else, embodied in the figure of the knife-grinder’s wife: her female kin and neighbours to whom she was going to return. If Mary Toft was not ‘telling the truth’ about Ann Toft’s role in the hoax, there was nevertheless something remarkable about implicating her mother-in-law to a Justice of the Peace. Mary Toft’s accusation against her mother-in-law – the eldest woman in the family and with whom she lived in close proximity – is itself suggestive of a fraught relationship. This reconstruction of Mary Toft’s embodied experience of pain and fear necessarily adjusts our interpretation of her as a scheming woman after money. Historians have treated Toft as responsible for the hoax, but she could not have done it alone and is unlikely to have done it willingly. Whether or not the stories in the confessions are true accounts of the events of autumn of 1726, Mary Toft’s experiences were those of a victim of female kin and neighbours exercising their authority over her body. As Lisa Cody notes, in 1726 ‘[m]edical-science had not yet conquered the reproductive female body’.^114^ And in this context some women retained considerable power not only over reproductive knowledge and other women’s bodies but the narratives of birth – hoax or otherwise – that could be told or enacted. It may have been a reluctance to encroach on this customary authority over truth telling that deterred the criminal prosecution of Toft and Howard – and notably *not* Ann Toft – for a misdemeanour. Several months after the rabbits first began to appear, these proceedings fizzled out, leaving Toft and the women around her to retreat back to the shadows.

## NOTES AND REFERENCES

1 *Weekly Journal or British Gazeteer*, Monday 10 Oct. 1726.

2 Nathaniel St. André, *A Short Narrative of an Extraordinary Delivery of Rabbets*, London, 1727, p. 23.

3 St. André, *Short Narrative*, pp. 23–4.

4 St. André, *Short Narrative*, p. 24.

5 St. André, *Short Narrative*, p. 25.

6 St. André, *Short Narrative*, pp. 25–6.

7 Dennis Todd, ‘Three Characters in Hogarth’s *Cunicularii* – and Some Implications’, *Eighteenth-Century Studies* 16: 1, fall 1982, pp. 26–46.

8 See, for example, Paul-Gabriel Boucé, ‘Imagination, Pregnant Women, and Monsters in Eighteenth-Century England and France’, in *Sexual Underworlds of the Enlightenment*, ed. George S. Rousseau and Roy Porter, Manchester 1987, pp. 86–100; Lisa Forman Cody, *Birthing the Nation: Sex, Science, and the Conception of Eighteenth-Century Britons*, Oxford, 2005, pp. 120–51; Marie-Hélène Huet, *Monstrous Imagination*, Cambridge MA and London, 1993.

9 A useful study of the case is Dennis Todd, *Imagining Monsters: Miscreations of the Self in Eighteenth-Century England*, Chicago and London, 1995. See also Cody, *Birthing the Nation*, pp. 120–51.

10 For a useful introduction see S. A. Seligman, ‘Mary Toft – the Rabbit Breeder’, *Medical History* 5: 4, 1961, pp. 349–60.

11 Jane Shaw, ‘Mary Toft, Religion and National Memory in Eighteenth-Century England’, *Journal for Eighteenth-Century Studies* 32: 3, 2009, pp. 321–8.

12 Philip K. Wilson, ‘Toft, Mary (*bap.* 1703, *d.* 1763)’, *Oxford Dictionary of National Biography*, Oxford University Press, 2004.

13 St André, *Short Narrative*, p. 23.

14 Mary Toft’s confession on 7 Dec. 1726, MS Hunter D324 (confession 1), Mary Toft’s confession on 8 Dec. 1726, MS Hunter D327 (confession 2) and Mary Toft’s confession on 12 Dec. 1726, MS Hunter D328 (confession 3) are kept with other documents relating to the case in papers belonging to Dr James Douglas, at Glasgow University Special Collections.

15 Lisa Cody, ‘“The Doctor’s in Labour; or, a New Whim Wham from Guildford”’, *Gender and History* 4: 2, 1992, p. 181.

16 Cody, *Birthing the Nation*, pp. 129–30; Cody, ‘The Doctor’s in Labour’, p. 191.

17 Todd, *Imagining Monsters*, pp. 4, 6.

18 For a useful survey see R. B. Outhwaite, ‘Age at Marriage in England from the Late Seventeenth to the Nineteenth Century’, *Transactions of the Royal Historical Society* 23, 1973, pp. 55–70.

19 Parish Registers for Godalming (St Peter and St Paul): Surrey History Centre. See also *The Parish Registers of Godalming: Indexes*, Godalming Museum.

20 Following his accounts of Toft’s explanations, St. André describes his actions on 23 November (which implies the account was taken before that date): St. André, *Short Narrative*, p. 28.

21 The case moved from the Westminster Sessions to the Court of King’s Bench, where it apparently collapsed. I explore this in more detail in Karen Harvey, ‘Rabbits, Whigs and Hunters: Rethinking Mary Toft’s Monstrous Births of 1726’, in preparation.

22 Richard Manningham, *An Exact Diary of What was Observ’d during a Close Attendance upon Mary Toft*, London, 1726, pp. 33–5. Lisa Cody mistakenly identifies Manningham’s summary as a condensed version of Mary Toft’s final confession, though the book was finished on the 7 December (the day the first confession was taken down). See Lisa Cody, ‘The Doctor’s in Labour’, p. 177.

23 A restatement of why Douglas cannot publish the confession, MS Hunter D329: Glasgow University Special Collections, recto.

24 Carlo Ginzburg, *The Cheese and the Worms: the Cosmos of a Sixteenth-Century Miller*, transl. John and Anne Tedeschi (1976), London, 1980, p. 59.

25 John Arnold, ‘The Historian as Inquisitor: the Ethics of Interrogating Subaltern Voices’, in *Rethinking History* 2: 3, 1998, pp. 379–86; François Soyer, *Ambiguous Gender in Early Modern Spain and Portugal: Inquisitors, Doctors and the Transgression of Gender Norms*, Leiden and Boston, 2012, pp. 12–14.

26 Sara Heller Mendelson, ‘“To Shift for a Cloak”: Disorderly Women in the Church Courts’, in *Women and History: Voices of Early Modern England*, ed. Valerie Frith, Toronto, 1995, p. 6.

27 Frances E. Dolan, *True Relations: Reading, Literature, and Evidence in Seventeenth-Century England*, Philadelphia, 2013, p. 117. See also pp. 56–86, 112–17.

28 Manningham, *An Exact Diary*, p. 32.

29 For example, Diane Purkiss, ‘Losing Babies, Losing Stories: Attending to Women’s Confessions in Scottish Witch-trials’, in *Culture and Change: Attending to Early Modern Women*, ed. Margaret Mikesell and Adele F. Seeff, Newark DE, 2003, pp. 143–60; Judith Bonzol, ‘The Medical Diagnosis of Demonic Possession in an Early Modern English Community’, *Parergon* 26: 1, 2009, pp. 129–30; Richard Raiswell, ‘Faking it: a Case of Counterfeit Possession in the Reign of James I’, *Renaissance and Reformation/Renaissance et Réforme* 35: 3, 1999, pp. 29–48.

30 Manningham, *An Exact Diary*, p. 26.

31 MS Hunter D324, 1r.

32 MS Hunter D327, 7r.

33 MS Hunter D324, 13r

34 MS Hunter D327, 6r.

35 Fair copy of Mary Toft’s confession, MS Hunter 325: Glasgow University Special Collections, 4r–4v.

36 MS Hunter D324, 8r.

37 Fair copy of Mary Toft’s confession, 1v.

38 Cody, ‘The Doctor’s in Labour’, p. 182.

39 Aldert Vrij, *Detecting Lies and Deceit: Pitfalls and Opportunities *(2000), Chichester, 2008, pp. 209–2. Mary Toft’s confessions meet at least fourteen of the nineteen criteria.

40 Vrij, *Detecting Lies and Deceit*, pp. 214–17.

41 Purkiss, ‘Losing Babies, Losing Stories’, p. 147.

42 Ginzburg, *The Cheese and the Worms*, p. 59. See Gary Schneider, ‘Affecting Correspondences: Body, Behavior, and the Textualization of Emotion in Early Modern English Letters’, *Prose Studies *23: 3, 2000, pp. 31–62, for a useful analysis of bodily substitution in writing.

43 Examples of early modern historians who urge this approach include Laura Gowing, *Common Bodies: Women, Sex and Reproduction in Seventeenth-Century England*, New Haven, 2003; Lyndal Roper, ‘Beyond Discourse Theory’, *Women’s History Review* 19: 2, 2010, pp. 307–19; Dror Wahrman, ‘Change and the Corporeal in Seventeenth- and Eighteenth-Century Gender History: Or, Can Cultural History Be Rigorous?’, *Gender & History* 20: 3, 2008, p. 599.

44 Monique Scheer, ‘Are Emotions a Kind of Practice (and Is That What Makes Them Have a History)? A Bourdieuian Approach to Understanding Emotion’, *History and Theory* 51: 2, 2012, p. 218.

45 MS Hunter D324, 1r.

46 MS Hunter D324, 1r.

47 MS Hunter D327, 1r.

48 St André, *A Short Narrative*, pp. 23–6.

49 Peter Brooks, *Troubling Confessions: Speaking Guilt in Law and Literature*, Chicago and London, 2000; Robert B. Shoemaker, ‘Print and Female Voice: Representations of Women’s Crime in London, 1690–1735’, *Gender & History* 22: 1, 2012, pp. 75–91.

50 Dolan, *True Relations*, throughout.

51 Didier Fassin and Richard Rechtman, *The Empire of Trauma: an Inquiry into the Condition of Victimhood* (Paris, 2007), transl. Rachel Gomme, Princeton and Oxford, 2009, p. 281.

52 Louise DeSalvo, *Writing as a Way of Healing: How Telling Our Stories Transforms Our Lives*, London, 1999, pp. 181–7.

53 See, for example, MS Hunter D328, 2v. Leah Astbury, ‘Delivered: After Birth Experiences in Seventeenth-Century England’, paper given to the conference ‘Birth: personal stories to population policies’, University of Leeds, 18–19 September 2014.

54 See Diane Purkiss, *The Witch in History: Early Modern and Twentieth Century Representations*, New York, 1996, p. 153.

55 Thomas Howard, surgeon of Guildford, described that Ahlers had taken animal parts from Toft’s uterus. See St André, *Short Narrative*, pp. 39–40.

56 St. André, *Short Narrative*, p. 8.

57 St. André, *Short Narrative*, p. 19.

58 On pain as sensation and perception see Jennifer Corns, ‘The Inadequacy of Unitary Characterizations of Pain’, *Philosophical Studies: an International Journal for Philosophy in the Analytic Tradition* 169: 3, 2014, pp. 355–78; Grant R. Gillett, ‘The Neurophilosophy of Pain’, *Philosophy* 66, April 1991, pp. 191–206; Ronald Melzack and Patrick D. Wall, *The Challenge of Pain*, 1982, Harmondsworth, 1996, pp. 15–33.

59 Melzack and Wall, *Challenge of Pain*, p. 41.

60 Melzack and Wall, *Challenge of Pain*, p. 43.

61 Jan Frans van Dijkhuizen and Karl A. E. Enenkel, ‘Introduction: Constructions of Physical Pain in Early Modern Culture’, in *The Sense of Suffering: Constructions of Physical Pain in Early Modern Culture*, ed. van Dijkhuizen and Enenkel, Leiden, 2008, pp. 2–3.

62 MS Hunter D324, 2r.

63 MS Hunter D324, 4r.

64 MS Hunter D324, 9r; MS Hunter D327, 3r; MS Hunter D328, 1r.

65 MS Hunter D324, 10r; MS Hunter D328, 3v.

66 MS Hunter D328, 3v.

67 Even after the hoax is exposed, Manningham acknowledges her pains were real. Manningham, *An Exact Diary*, pp. 35–6.

68 MS Hunter D324, 5r.

69 Van Dijkhuizen and Enenkel, ‘Introduction: Constructions of Physical Pain’, p. 8; Lisa Wynne Smith, ‘“An Account of an Unaccountable Distemper”: the Experience of Pain in Early Eighteenth-Century England and France’, *Eighteenth-Century Studies* 41: 4, 2008, pp. 459–80.

70 MS Hunter D324, 11r. St. André, *Short Narrative*, p. 29.

71 MS Hunter D327, 1r. For an earlier use of this metaphor by her, see St André, *Short Narrative*, p. 29.

72 Sharon Howard, ‘Imagining the Pain and Peril of Seventeenth-Century Childbirth: Travail and Deliverance in the Making of an Early Modern World’, *Society for the Social History of Medicine* 16: 3, 2003, p. 377.

73 MS Hunter D328, 2v.

74 Howard, ‘Imagining the Pain and Peril’, pp. 367–82.

75 William E. Burns, *An Age of Wonders: Prodigies, Politics, and Providence in England, 1657–1727*, p. 187 and throughout.

76 Philip M. Soergel, ‘Agnes Bowker’s Cat, the Rabbit Woman of Godalming, and the Shifting Nature of Portents in Early Modern Europe’, *Studies in Medieval & Renaissance History*, 3rd ser., 4, 2007, p. 282. On the politics of the Toft case, see also Harvey, ‘Rabbits, Whigs and Hunters’.

77 Elaine Scarry, *The Body in Pain*: *the Making and Unmaking of the World*, Oxford, 1985.

78 MS Hunter D328, 2r.

79 MS Hunter D327, 9r.

80 MS Hunter D328, 11v; MS Hunter D327, 4r.

81 St André, *A Short Narrative*, pp. 9, 10. See also p. 14 for another use of ‘easy’.

82 MS Hunter D327, 8r.

83 Scheer, ‘Are Emotions a Kind of Practice’; Olivia Weisser, ‘Grieved and Disordered: Gender and Emotion in Early Modern Patient Narratives’, *Journal of Medieval and Early Modern Studies* 43: 2, 2013, pp. 247–73.

84 St. André, *Short Narrative*, p. 12.

85 Manningham, *Exact Diary*, p. 25.

86 St. André, *A Short Narrative*, p. 30.

87 *The Several Depositions of Edward Costen, Richard Stedman, John Sweetapple, Mary Peytoe, Elizabeth Mason, and Mary Costen; relating to the Affair of Mary Toft*, London, 1727, p. 19.

88 Dana Y. Rabin, ‘Searching for the Self in Eighteenth-Century English Criminal Trials, 1730–1800’, *Eighteenth-Century Life* 27: 1, 2003, p. 86.

89 MS Hunter D324, 2r

90 MS Hunter D324, 2r; MS Hunter D327, 9r; MS Hunter D328, 2v.

91 MS Hunter D327, 8r.

92 MS Hunter D328, 1r.

93 MS Hunter D328, 2r.

94 MS Hunter D328, 1r, 3r.

95 St. André, *Short Narrative*, p. 8.

96 Manningham, *An Exact Description*, p. 11.

97 MS Hunter D324, 2r; MS Hunter D328, 2v; MS Hunter D328, 1r, 3r.

98 MS Hunter D324, 3r.

99 MS Hunter D328, 1r.

100 *The Parish Registers of Godalming: Indexes*. See burials for Widow Smith December 1728, Mrs Tickner April 1737 and Mary Edsell September 1762. Neither the Archdeaconry of Surrey Muniment book for April 1708–December 1720 (DW/OB/2) nor that for February 1720/1–January 1730/1 (DW/OB/3), held at the London Metropolitan Archives, lists Ann Toft.

101 MS Hunter D327, 9r.

102 Gowing, *Common Bodies*, p. 50. For other works on the nature of the lying-in see Linda A. Pollock, ‘Childbearing and Female Bonding in early modern England’, *Social History* 22: 3, 1997, pp. 286–306; Adrian Wilson, ‘Participant or Patient: Seventeenth-Century Childbirth from the Mother’s Point of View’, in *Patients and Practitioners: Lay Perceptions of Medicine in Pre-Industrial Society*, ed. Roy Porter, Cambridge, 1985, pp. 129–44; Adrian Wilson, ‘The Ceremony of Childbirth and its Interpretation’, in *Women as Mothers in Pre-Industrial England: Essays in Memory of Dorothy Maclaren*, ed. Valerie A. Fildes, London, 1990, pp. 68–107.

103 James A. Sharpe, ‘Witchcraft and Women in Seventeenth-Century England’, *Continuity and Change* 6: 2, 1991, pp. 179–99. See also Garthine Walker, *Crime, Gender and Social Order in Early Modern England*, Cambridge, 2003, where she argues that ‘women’s violence was a manifestation of contests for feminine superiority’ (p. 98).

104 MS Hunter D324, 5r.

105 MS Hunter D324, 6r.

106 MS Hunter D324, 5v.

107 Cody, *Birthing the Nation*, p. 129.

108 Purkiss, ‘Losing Babies, Losing Stories’, pp. 143–60. Jonathan B. Durrant, *Witchcraft, Gender and Society in Early Modern Germany*, Leiden, 2007. Lyndal Roper, ‘“Evil Imaginings and Fantasies”: Child-Witches and the End of the Witch Craze’, *Past & Present* 167: 1, 2000, pp. 107–39, is a useful comparison which explores an Augsburg case of 1723–6, a moment ‘on the cusp of the Enlightenment’ (p. 108).

109 Louise Jackson, ‘Witches, Wives and Mothers: Witchcraft Persecution and Women’s Confessions in Seventeenth-Century England’, *Women’s History Review* 4: 1, 1995, p. 74 and throughout; Lyndal Roper, *Oedipus and the Devil: Witchcraft, Sexuality and Religion in Early Modern Europe*, London, 1994, pp. 199–225.

110 Walker, *Crime, Gender and Social Order*, p. 97.

111 Linda A. Pollock, ‘Childbearing and Female Bonding’, *Social History *22: 3, 1997, pp. 290–1; Linda A. Pollock, ‘Embarking on a Rough Passage: the Experience of Pregnancy in Early Modern Society’, in *Women as Mothers in Pre-Industrial England*, ed. Fildes, pp. 47–9. Herman Roodenburg, ‘The Maternal Imagination: the Fears of Pregnant Women in Seventeenth-Century Holland’, *Journal of Social History* 21: 4, 1988, pp. 701–16.

112 MS Hunter D324, 9r.

113 Manningham, *Exact Diary*, p. 14.

114 Cody, ‘The Doctor’s in Labour’, p. 190.

